# Safety and effectiveness of diazepam nasal spray in male and female patients: Post hoc analysis of data from a phase 3 safety study

**DOI:** 10.1002/epi4.12907

**Published:** 2024-02-10

**Authors:** James W. Wheless, Robert Edward Hogan, Charles S. Davis, Enrique Carrazana, Adrian L. Rabinowicz

**Affiliations:** ^1^ Le Bonheur Children's Hospital University of Tennessee Health Science Center Memphis Tennessee USA; ^2^ Washington University in St. Louis St. Louis Missouri USA; ^3^ CSD Biostatistics Oro Valley Arizona USA; ^4^ Neurelis, Inc. San Diego California USA; ^5^ University of Hawaii John A. Burns School of Medicine Honolulu Hawaii USA

**Keywords:** acute repetitive seizures, benzodiazepines, diazepam, gender, intranasal, sex

## Abstract

Sex differences in drug pharmacokinetics include variations in the expression of the cytochrome P450 enzymes, which are involved in the metabolism of benzodiazepines. It is unclear whether sex influences outcomes associated with intranasally administered drugs. A post hoc analysis of sex differences was conducted to evaluate the effectiveness and safety of diazepam nasal spray, which included examining changes in the number of days between seizure clusters over time (SEIzure interVAL [SEIVAL]). Diazepam nasal spray is approved for acute treatment of seizure clusters in patients with epilepsy aged ≥6 years. Data from a phase 3 safety study were used to determine the proportion of second doses used within 24 h (ie, a proxy for effectiveness) and SEIVAL. Adverse events were recorded. Of 163 treated patients, 89 were female, and 74 were male. Approximately 16% of both sexes self‐administered the study drug. A slightly higher proportion of seizure clusters was treated with a second dose in female (14.7%) than male (9.4%) patients. SEIVAL increased significantly and substantially over a year for all patients. The safety profile was generally similar between the sexes. These results suggest that potential sex differences in benzodiazepine pharmacokinetics do not meaningfully influence outcomes associated with diazepam nasal spray.

**Plain Language Summary:**

Some drugs may have differences in absorption and metabolism between genders that could translate into differences in safety and effectiveness. This safety study looked at diazepam nasal spray for treating seizure clusters in patients at least 6 years old. It found that safety was about the same for females and males. For both groups, most clusters stopped after only 1 dose of the drug, and the time between treated clusters got longer over a year.

**Key points**

- The influence of sex on outcomes associated with intranasal rescue therapy for seizure clusters has not been fully characterized.
- A post hoc analysis of data from a long‐term safety study of diazepam nasal spray for seizure clusters evaluated outcomes by sex.
- Initial dose effectiveness was high for both sexes and mean time between treated seizure clusters increased in a similar pattern over time.
- Adverse event rates were also similar between male and female patients.
- These results suggest that intermittent use of diazepam nasal spray did not meaningfully influence outcomes between females and males.

## 1 INTRODUCTION

Selecting the appropriate antiseizure medication (ASM) for an individual patient with epilepsy requires consideration of both medication‐ and patient‐related variables.^1^ One demographic variable that potentially should be considered is the patient's biological sex.^1^

Clinically relevant sex differences in the effects of medications have been discussed in the literature. In a review of a database including 400 drug substances for multiple therapeutic areas, 20% were found to have clinically relevant sex differences, mainly regarding efficacy and adverse events.^2^ Pharmacokinetic (PK) parameters have been shown to be affected by sex for such drugs as metformin, cannabidiol, aspirin, warfarin, and digoxin, and differences in adverse events and PK by sex have been shown for cardiovascular drugs.^3^, ^4^, ^5^, ^6^, ^7^

Regarding drug PK, sex differences have been found in association with bioavailability, distribution, metabolism, and elimination.^8^ Sex differences in drug metabolism include differentiation in the expression of key cytochrome (CY) P450 enzymes important for benzodiazepine metabolism.^9^ CYP3A substrates (eg, benzodiazepines) have been associated with sex‐related differences in PK (eg, 20%–30% increased hepatic clearance for female vs. male patients; higher values of weight‐normalized clearance in female patients).^10^, ^11^

Benzodiazepines are the cornerstone of rescue therapies for seizure clusters,^12^ which may occur in patients with epilepsy despite a daily regimen of ASMs.^13^ Diazepam nasal spray (Valtoco®) is approved for acute treatment of intermittent, stereotypic episodes of frequent seizure activity (ie, seizure clusters, acute repetitive seizures) that are distinct from a patient's usual seizure pattern in patients with epilepsy aged ≥6 years.^14^

The influence of sex on outcomes associated with intranasal rescue therapy for seizure clusters has not been fully characterized. Covariate analyses indicate that PK and analyses of effectiveness and safety appear to be generally similar between the sexes.^14^, ^15^ This post hoc analysis examines whether sex‐related PK differences may have an impact on the effectiveness and safety of diazepam nasal spray, including the duration in days between seizure clusters (SEIzure interVAL [SEIVAL]) over time, in patients with seizure clusters.

## 2 METHODS

Data were obtained from patients aged 6–65 years in the safety population of a phase 3, long‐term, open‐label, repeat‐dose safety study of diazepam nasal spray.^16^ The study had a 21‐day screening phase, baseline assessment period (within 72 h prior to day 0), and 12‐month treatment period, with study visits at day 30 and every 60 days thereafter. Enroled patients had a diagnosis of focal or generalized onset epilepsy with motor seizures or seizures with clear alteration of awareness and, in the investigator's opinion, might also need benzodiazepine treatment for seizure control ≥6 times a year on average despite a stable regimen of daily ASM. History of status epilepticus or seasonal allergies/rhinitis was permitted, and concomitant use of benzodiazepines (eg, clobazam) was not restricted. The study's full methodology was published previously.^16^ The operational definition for seizure clusters used a 24‐h period.^17^ Patients received 5‐, 10‐, 15‐, or 20‐mg doses (based on age and weight).^16^ Seizure timing and drug administration were recorded in a patient diary.^16^

### 2.1 Second dose analysis

Per protocol and investigator guidance, second doses of diazepam nasal spray could be administered 4–12 h after the first dose if needed to control a seizure cluster.^16^ Investigators could adjust doses as clinically warranted, including adjusting the timing of second doses.^17^ The proportion of seizure clusters for which second doses were given within 24 h of the first was used as a proxy for effectiveness, demonstrating whether the seizure clusters were controled by a single dose.^16^

### 2.2 SEIVAL analysis

Patient diary data were used to examine the recurrence of treated seizure clusters using the number of days between doses of diazepam nasal spray.^18^ SEIVAL was defined as the time between 2 treated seizure clusters. In a previous post hoc analysis, SEIVAL was used to test the potential impact of intermittent rescue therapy on seizure clusters over time in the safety population of the phase 3 safety study. Change in SEIVAL over time was determined using 90‐day periods; 4 periods corresponded to 360 days, which was similar to the 12‐month treatment period of the study. Patients with SEIVALs in all 4 periods (Periods 1–4, consistent cohort) were the focal point of the analysis as a way to address potential confounding due to a variable cohort over time.^18^

In the present post hoc analysis, SEIVAL was evaluated in male and female patients to better understand this metric. Two versions of the analysis were performed. Initially, all doses of diazepam nasal spray were included in the analysis (ie, retreatments for a seizure cluster were included). In a second version of the analysis, second doses administered within 24 h of the first dose were excluded to eliminate retreatments to measure time between seizure clusters rather than doses. Pearson's chi‐square test (2‐sided) was used to compare proportions between male and female patients. Two‐sided 2‐sample *t*‐tests were used to assess statistically significant differences between male and female patients in mean SEIVAL. Two‐sided paired *t* tests were used for within‐sex comparisons.

### 2.3 Safety

Rates of treatment‐emergent adverse events (TEAEs), serious TEAEs, and treatment‐related TEAEs were recorded.

## 3 RESULTS

Overall results for the phase 3 study of diazepam nasal spray were published previously.^16^ Of the 175 patients enroled in the study, 163 (female: 89 [54.6%]; male: 74 [45.4%]) were treated with ≥1 dose of diazepam nasal spray.^16^ Demographic characteristics, mean doses per month, and mean seizure clusters per patient were similar between the sexes (Table 1).

**TABLE 1:** Patient characteristics (*N* = 163).

	Male patients (*n* = 74)	Female patients (*n* = 89)
Age group, *n* (%)		
6–11 years	19 (25.7)	26 (29.2)
≥12 years	55 (74.3)	63 (70.8)
Doses per month, mean (SD)	2.1 (1.0)	2.5 (1.8)
Seizure clusters, mean per patient	20.8	26.0
Self‐administrators, *n* (%)	12 (16.2)	15 (16.9)
Developmental epileptic encephalopathies (*n* = 64), *n* (%)	29 (45.3)	35 (54.7)
Rett syndrome (*n* = 16)	0	16 (100)
Lennox–Gastaut syndrome (*n* = 9)	5 (55.6)	4 (44.4)
Dravet syndrome (*n* = 7)	6 (85.7)	1 (14.3)

Approximately 16% of both sexes reported self‐administering diazepam nasal spray. Self‐administration was reported on a study exit survey by a total of 27 of the 163 patients (15 female and 12 male).^19^

### 3.1 Use of second dose of diazepam nasal spray

In the overall safety population, second doses were used for 485 of 3853 seizure clusters (12.6%).^16^ Among the 79 patients who administered second doses, 40 (50.6%) were female and 39 (49.4%) were male.^17^ Among the 84 patients not receiving second doses, 49 (55.1%) were female and 35 (47.3%) were male (*p* = 0.32, Pearson's chi‐square test 2‐sided).

The means of individual patients' percentages of seizure clusters for which second doses were administered were similar between female patients (*n* = 89; mean [SD], 7.9% [12.0%]) and male patients (*n* = 74; 8.3% [15.1%]) (*p* = 0.74, 2‐sided, 2‐sample *t* test). The overall proportion of seizure clusters for which second doses were administered was slightly higher across the full female cohort (341 of 2316 seizure clusters [14.7%]) than across the full male cohort (144 of 1537 seizure clusters [9.4%]). All male patients and most female patients received <20 second doses during the study; 4 female patients received >20 second doses, including 2 patients who received >50 second doses (24, 24, 61, and 82 second doses) during the study.

### 3.2 SEIVAL

Overall in the safety population, 76 patients in the consistent cohort had ≥1 SEIVAL in each of the first four 90‐day periods.^18^ Of these patients, 46 (60.5%) were female and 30 (39.5%) were male.

**FIGURE 1:**
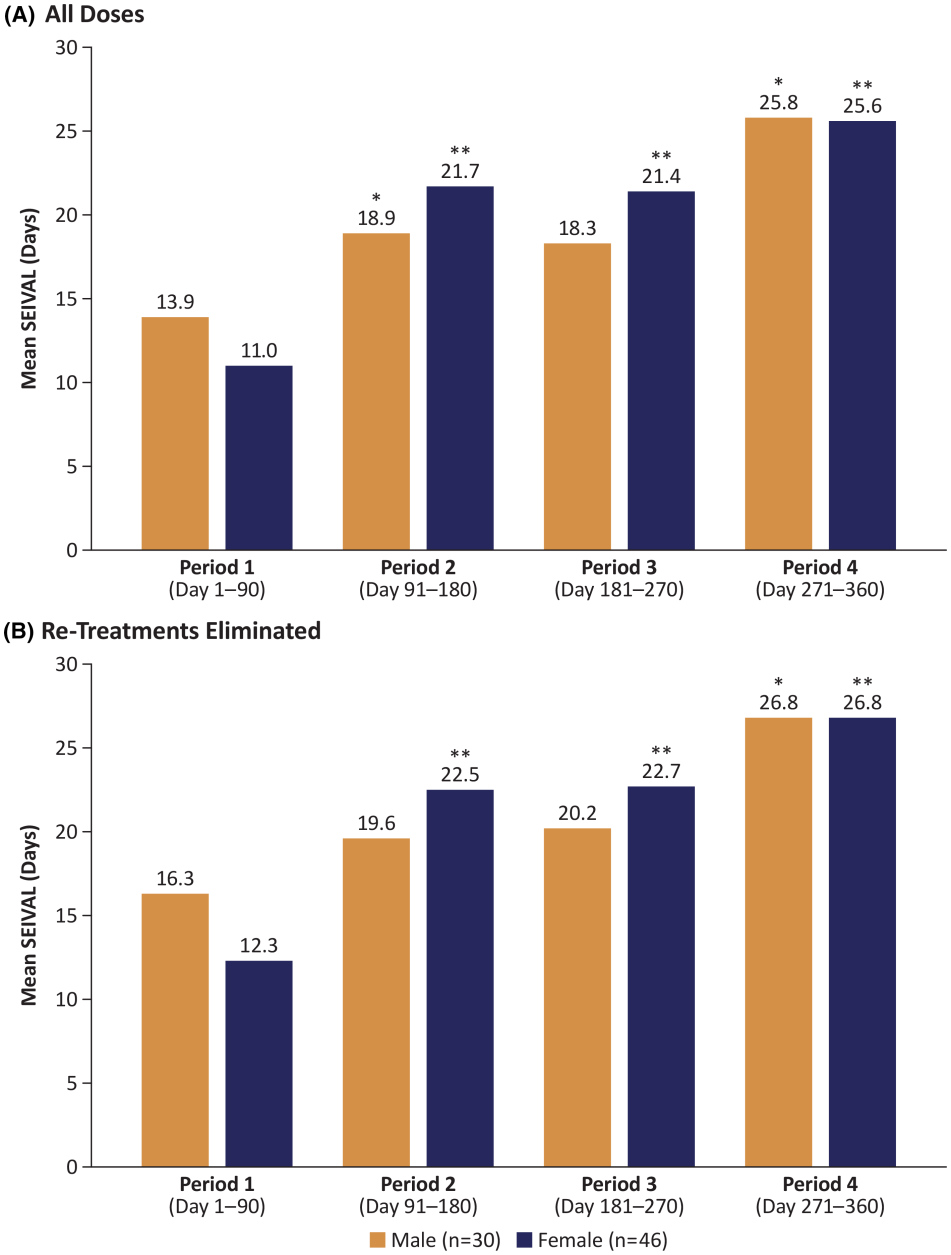
Mean SEIzure InterVAL (SEIVAL) between seizure clusters in male and female patients across 360 days (A) for all doses and (B) with re‐treatments eliminated. *p < 0.05 compared with Period 1, **p < 0.01 compared with Period 1.

In the SEIVAL analysis by sex that included all doses (Figure 1A), female patients had a slightly larger increase (14.6 days) in mean SEIVAL from Period 1 (11.0 days) to Period 4 (25.6 days) (*p* < 0.01). Significant increases also were seen from Period 1 to Period 2 and from Period 1 to Period 3 (*p* < 0.01). In male patients, mean SEIVAL increased 11.9 days from 13.9 days at Period 1 to 25.8 days at Period 4 (*p* < 0.05).

Similarly, in the analysis by sex that excluded second doses as retreatments of seizure clusters (Figure 1B), female patients had a slightly larger increase (14.5 days) in mean SEIVAL from Period 1 (12.3 days) to Period 4 (26.8 days) (*p* < 0.01). Significant increases also were seen from Period 1 to Period 2 and from Period 1 to Period 3. In male patients, mean SEIVAL increased 10.5 days from 16.3 days in Period 1 to 26.8 days in Period 4 (*p* < 0.05).

### 3.3 Safety

The safety profile of diazepam nasal spray was generally similar between female and male patients (Table 2). Treatment‐related TEAEs were reported in 18.0% of female and 18.9% of male patients. None of the patients reported respiratory depression.^16^ No serious adverse events reported in the study were considered treatment related.^16^ One discontinuation due to a TEAE (major depression) occurred in a female patient and one death (sudden unexpected death in epilepsy) occurred in a male patient; neither was considered treatment related.^16^

**TABLE 2:** Safety characteristics (*N* = 163).

	Male patients (*n* = 74)	Female patients (*n* = 89)
TEAEs	59 (79.7)	75 (84.3)
Most common (>10%, either group)		
Seizure	15 (20.3)	16 (18.0)
Upper respiratory tract infection	10 (13.5)	10 (11.2)
Pyrexia	7 (9.5)	10 (11.2)
Influenza	4 (5.4)	9 (10.1)
Nasopharyngitis	12 (16.2)	8 (9.0)
Serious TEAEs	25 (33.8)	25 (28.1)
Treatment‐related TEAEs	14 (18.9)	16 (18.0)
Most common (>2% either group)		
Nasal discomfort	5 (6.8)	5 (5.6)
Cough	0 (0.0)	2 (2.2)
Rhinorrhea	0 (0.0)	2 (2.2)
Dysgeusia	1 (1.4)	2 (2.2)
Headache	2 (2.7)	2 (2.2)
Somnolence	2 (2.7)	1 (1.1)
Epistaxis	2 (2.7)	1 (1.1)
Rhinalgia	2 (2.7)	0 (0.0)
Discontinued due to TEAE^a^	0 (0.0)	1 (1.1)
Death^a^	1 (1.4)	0 (0.0)

Abbreviation: TEAE, treatment‐emergent adverse event.

^a^ Not considered treatment related.

## 4 DISCUSSION

In this analysis, outcomes by sex were generally similar regarding the effectiveness or safety profile of diazepam spray. Usage of second doses within 24 h of the initial dose was low for both sexes, demonstrating the effectiveness of a single dose to control a seizure cluster. SEIVAL increased in a similar pattern over time for both sexes, suggesting a potential impact of diazepam nasal spray. Rates of TEAEs were similar.

Sex differences in liver clearance have been demonstrated for such benzodiazepines as alprazolam, midazolam, and triazolam.^20^ Drug clearance may be modified by sex hormones in female patients through inhibition or induction of CYP enzymes.^21^ Clearance was not examined in the present analysis, but these results clarify that the effectiveness and safety of diazepam nasal spray did not differ between female and male patients as a treatment for seizure clusters. Notably, most patients with Dravet syndrome were male, and patients with Rett syndrome were female; the potential impact of these differences on the present results was not analyzed.

This analysis showed that self‐administration of diazepam nasal spray was feasible in an equivalent proportion of female and male patients. Self‐administration with diazepam nasal spray is supported when consistent with the prescribing information.^19^

The 79 patients using second doses in this study were evenly split between female and male patients; the difference was not statistically significant.^17^ Most patients who used second doses (82.3%) had exposure to diazepam nasal spray for a year or longer. Among patients using a second dose, there was a wide range of second doses used per patient (1–82), with a mean of 6.1 and 41.8% receiving a single second dose.^17^

In both versions of the SEIVAL analysis, days between seizure clusters doubled for female patients from Period 1 to Period 4, marking a significant increase; male patients had a slightly shorter increase that was also significant. Interestingly, although baseline SEIVALs differed by sex, SEIVALs in Period 4 were nearly identical. Hypotheses regarding potential causes for these increases in SEIVAL have been discussed in a previous publication, including biological factors (eg, altering underlying biology of clusters), behavioral change (eg, intentional or unintentional adherence), and regression to the mean.^18^

Currently, 3 rescue therapies are approved in the US for the treatment of acute seizure clusters: diazepam rectal gel, midazolam nasal spray, and diazepam nasal spray.^14^, ^15^, ^22^ Although outcomes by sex were similar in the present analyses of diazepam nasal spray, further analysis could help characterize potential smaller differences between the sexes among the rescue therapies.

This open‐label safety study had no control or comparator group, limiting its power; however, it evaluated a proven agent, diazepam. Diary use may have limiting factors, such as understanding instructions and completeness of entries. The analyses only included treated clusters; other types of seizures were not recorded.

## 5 CONCLUSIONS

In this post hoc analysis of data from a long‐term safety study, the effectiveness and safety of diazepam nasal spray were generally similar between female and male patients. Patients of both sexes demonstrated significant increases in mean SEIVAL, a potential outcome measure for rescue medication, between the beginning and end of the treatment period, showing similar patterns over time. These results suggest that potential sex differences in benzodiazepine PK do not meaningfully influence outcomes associated with intermittent use of diazepam nasal spray.

## AUTHOR CONTRIBUTIONS

Original study conceptualization and supervision: ALR, EC. Data collection: JWW, REH. Conceptualization of present analysis and data interpretation: All authors. All authors contributed to drafting, critically reviewing, and revising the manuscript and approved the submitted version.

## FUNDING INFORMATION

This study was funded by Neurelis, Inc. (San Diego, CA).

## CONFLICT OF INTEREST STATEMENT

Dr Wheless has served as an advisor or consultant for CombiMatrix; Eisai Inc.; GW Pharmaceuticals; Lundbeck, Inc.; Neurelis, Inc.; NeuroPace, Inc.; Supernus Pharmaceuticals, Inc.; and Upsher‐Smith Laboratories, Inc. He has served as a speaker or a member of a speakers bureau for Cyberonics, Inc.; Eisai Inc.; Lundbeck, Inc.; Mallinckrodt; Neurelis, Inc.; Supernus Pharmaceuticals, Inc.; and Upsher‐Smith Laboratories, Inc., and has received grants for clinical research from Acorda Therapeutics; GW Pharmaceuticals; Insys Therapeutics, Inc.; Lundbeck, Inc.; Mallinckrodt; Neurelis, Inc.; NeuroPace, Inc.; Upsher‐Smith Laboratories, Inc.; and Zogenix, Inc. Dr Hogan has received research support from UCB Pharmaceuticals; Neurelis, Inc.; and Biogen, and he is an advisor for Neurelis, Inc. Dr Davis is a consultant for Neurelis, Inc. Dr Carrazana is an employee of and received stock and stock options from Neurelis, Inc. Dr Rabinowicz is an employee of and has received stock options from Neurelis, Inc. We confirm that we have read the Journal's position on issues involved in ethical publication and affirm that this report is consistent with those guidelines.

## ETHICS STATEMENT

Data for this analysis were used post hoc from the phase 3 study of diazepam nasal spray; the original study protocol, informed consent form, and other relevant study documentation were approved by ethics committees or institutional review boards at each site before study initiation.

## CLINICAL TRIAL REGISTRATION

Data for this analysis were used post hoc from the phase 3 study of diazepam nasal spray, NCT02721069.

## Data Availability

All relevant data are within the article.
